# Supplementary material for: Cross-property deep transfer learning framework for enhanced predictive analytics on small materials data
Source: Nat Commun. 2021 Nov 15;12:6595. doi: 10.1038/s41467-021-26921-5 (PMC8594437; doi:10.1038/s41467-021-26921-5)
Supplement: Supplementary file 1 — Supplementary Information [file 41467_2021_26921_MOESM1_ESM.pdf]

Supplementary Information:
Cross-property deep transfer learning framework for enhanced predictive analytics on small materials data

Vishu Gupta[1], Kamal Choudhary[2,3], Francesca Tavazza[2], Carelyn Campbell[2], Wei-keng Liao[1], Alok Choudhary[1], Ankit Agrawal[1*]

[1]*Department of Electrical and Computer Engineering, Northwestern University*

[2]*Materials Measurement Laboratory, National Institute of Standards and Technology, Gaithersburg, MD,20899, U.S.A.*

[3]*Theiss Research, La Jolla, CA, 92037, U.S.A.*

*Correspondence to Ankit Agrawal (email: ankitag@eecs.northwestern.edu).

Supplementary Table 1: Data Size for each of the materials property used to train Source Model

Dataset	Property	Abbreviation	Size
OQMD	Formation Energy_per_atom (eV)	Deltae	341450
OQMD - JARVIS	Formation Energy (eV/atom)	Deltae	321140
	Band Gap (eV)	Bandgap	321140
	Energy_per_atom (eV/atom)	Total	321140
	Stability	Stability	302313
	Magnetic Moment (μ_B)	Magmom	183874
	Volume_pa (Å^3)	Volume	321140

Supplementary Table 2: Data Size and Category for each of the materials property used to train the Target Model

Dataset	Property	Abbreviation	Category	Size
JARVIS	Kpoints Length Unit (Å)	KLU	Electronic-Structure	28171
	Kpoints Array Average (Å)	KAA	Electronic-Structure	28163
	Bandgap Optb88vdw (eV)	BgOptb	Conductivity	28155
	Formation Energy (eV/atom)	Deltae	Stability	28108
	Encut (eV)	Encut	ElectroMagnetism	28056
	Ehull (eV/atom)	Ehull	Stability/Decay	27297
	Magmom Oszicar (μ_B)	MagOszi	Magnetism	25844
	Magmom Outcar (μ_B)	MagOut	Magnetism	25357
	Eps Refractive Index (x)	Eps	Photo Optics	25150
	P Powerfact (Wm_1K_1)	PPf	Electricity	16760
	N Powerfact ($Wm\ 1K\ 1$)	NPf	Electricity	16763
	P Effective Masses 300K Average (kg)	PEM300K	Conductivity	16250
	N Effective Masses 300K Average (kg)	NEM300K	Conductivity	16250
	P Seebeck ($\mu V/K$)	PSb	Thermoelectric	14439
	N Seebeck ($\mu V/K$)	NSb	Thermoelectric	14144
	Meps Refractive Index (x)	Meps	Photo Optics	11349
	Max (Phonon) Mode (cm^{-1})	MaxM	Mechanics	10963
	Min (Phonon) Mode (cm^{-1})	MinM	Mechanics	10930
	Elastic Tensor C11 (GPa)	ETC11	Elasticity	10839
	Elastic Tensor C12 (GPa)	ETC12	Elasticity	10759
	Elastic Tensor C13 (GPa)	ETC13	Elasticity	10846
	Elastic Tensor C22 (GPa)	ETC22	Elasticity	10832
	Elastic Tensor C33 (GPa)	ETC33	Elasticity	10856
	Elastic Tensor C44 (GPa)	ETC44	Elasticity	9986
	Elastic Tensor C55 (GPa)	ETC55	Elasticity	9755
	Elastic Tensor C66 (GPa)	ETC66	Elasticity	9739
	Bulk Modulus KV (GPa)	Bulk	Elasticity	10743
	Shear Modulus GV (GPa)	Shear	Elasticity	10209
	Bandgap MBJ (eV)	BgMbj	Conductivity	7296
	Spillage (Å^{-1})	Spillage	Spin-Orbit Coupling	3866
	SLME (%)	SLME	Photovoltic Efficiency	3006
	Max Ir Mode (cm^{-1})	MaxIrM	Electromagnetic Radiation	2302
	Min Ir Mode (cm^{-1})	MinIrM	Electromagnetic Radiation	2268
	Dfpt Piezo Max Dielectric Electronic (ε_{11})	PMDiEl	Electric Potential	2126
	Dfpt Piezo Max Dielectric (ε_{11})	PMDi	Electric Potential	2126
	Dfpt Piezo Max Dielectric Ioonic (ε_{11})	PMDiIo	Electric Potential	2126
	Dfpt Piezo Max Eij (cm^{-2})	PMEij	Electric Potential	1123
	Dfpt Piezo Max Dij (cm^{-2})	PMDij	Electric Potential	689
	Exfoliation Energy (eV/atom)	Exfoli	Mechanical Energy Cost	557

Supplementary Table 3: Modified target properties and their description

Notation	Description
Kpoints Array Average	Average of the each entry in the Kpoints Array
Eps Refractive Index	Square root of average of Epsx, Epsy, Epsz
Meps Refractive Index	Square root of average of Mepsx, Mepsy, Mepsz
Max Mode	Maximum value of the Modes Array
Min Mode	Minimum value of the Modes Array
P Effective Masses 300K Average	Average of the P type Effective Masses at 300K
N Effective Masses 300K Average	Average of the N type Effective Masses at 300K
Elastic Tensor C11	Elastic Tensor (ET) at (1,1) of the ET array
Elastic Tensor C12	Elastic Tensor (ET) at (1,2) of the ET array
Elastic Tensor C13	Elastic Tensor (ET) at (1,3) of the ET array
Elastic Tensor C22	Elastic Tensor (ET) at (2,2) of the ET array
Elastic Tensor C33	Elastic Tensor (ET) at (3,3) of the ET array
Elastic Tensor C44	Elastic Tensor (ET) at (4,4) of the ET array
Elastic Tensor C55	Elastic Tensor (ET) at (5,5) of the ET array
Elastic Tensor C66	Elastic Tensor (ET) at (6,6) of the ET array

Supplementary Table 4: Hyper-parameters used for ML algorithms

ML algorithm	Hyper-parameters	# models
Adaboost	Default	1
Elastic Net	Default	1
Linear Regression	Default	1
SGD Regression	Penalty:{'l1', 'l2', 'elastic net', 'none'} x L1_ratio:{0.01, 0.02, 0.10, 0.20, 0.50, 0.80} x Fit_intercept:{False / True}	48
Ridge	Solver:{'svd', 'cholesky', 'lsqr', 'sparse_cg', 'sag', 'saga'} x Normalize:{False / True} x Fit_intercept:{False / True}	24
Support Vector Machine	C:{1, 2, 5, 10, 100, 1000} x Kernel: {'linear', 'poly', 'rbf'}	18
K-Neighbors	N_neighbors:{2, 5, 10, 100} x Weights:{uniform / distance} x algorithm:{'ball_tree', 'kd_tree', 'brute'}	24
Desicion Tree	Max_depth:{2, 5, 10, 100, 1000} x Max_features:{'none', 'sqrt', 'log2'}	15
Extra Tree	Max_depth:{10, 11, 12, 13, 14, 15, 16, 17, 18, 19, 20, 21, 22, 23, 24, 25}	16
Bagging	Default	1
Random Forest	Min_sample_split:{5, 10, 15, 20} x N_estimators:{100, 150, 200} x Max_depth:{10, 25}	24
Lasso	Alpha:{'0.01', '0.02', '0.05', '0.10', '0.20', '0.05', '1.00', '2.00', '5.00', '10.00'}	10

Supplementary Table 5: Prediction performance benchmarking for the prediction task of "Multi Target Transfer Learning" using only formation energy as the source property. The table shows the validation MAE of different target materials properties for SC and TL models. All the model inputs are based on EF.

Property	Base	Scratch (SC) Models		Transfer Learning (TL) with Deltae as source property				
		SC: ML(EF)	SC:DL(EF)	TL: ML(FeatExtr)	TL:DL(FeatExtr)	TL:FineTune	TL:ModFineTune	TL:Freezing
**KLU**	19.03	12.01	11.83	**11.35**	11.37	11.66	11.53	15.24
**KAA**	5.027	2.957	2.972	2.857	**2.855**	2.895	2.899	3.971
**BgOptb**	0.941	0.314	0.246	0.283	0.217	0.215	**0.211**	0.569
**Deltae**	0.861	0.314	0.141	0.162	0.126	**0.120**	0.124	0.234
**Encut**	244.9	**83.05**	88.89	105.6	86.72	92.17	92.38	187.0
**Ehull**	0.130	0.068	0.057	0.064	0.053	**0.050**	0.052	0.069
**MagOszi**	1.201	0.434	0.390	0.489	0.354	**0.341**	0.361	0.727
**MagOut**	1.251	0.459	0.403	0.466	0.359	0.358	**0.343**	0.806
**Eps**	3.734	1.525	1.418	1.431	**1.263**	1.285	1.286	2.782
**PPf**	649.6	514.8	514.9	494.7	**477.2**	488.8	487.6	596.6
**NPf**	647.1	555.4	531.4	516.1	**496.5**	516.2	507.5	601.8
**PEM300K**	1.972	1.277	1.225	1.208	1.190	**1.157**	1.208	1.715
**NEM300K**	1.972	1.277	1.235	1.208	**1.135**	1.159	1.221	1.717
**PSb**	165.9	61.06	59.64	61.23	**53.02**	54.03	54.36	104.4
**NSb**	111.8	57.70	54.23	56.73	**47.71**	50.16	50.47	84.11
**Meps**	4.831	2.143	2.010	2.064	**1.840**	1.874	1.843	3.740
**MaxM**	297.5	66.02	67.41	79.84	61.95	65.23	**59.05**	231.7
**MinM**	37.08	22.60	21.25	22.53	**18.90**	20.52	20.46	23.23
**ETC11**	78.65	40.11	35.90	34.88	**32.93**	35.09	33.18	74.80
**ETC12**	43.26	18.66	17.17	16.58	**15.40**	16.07	16.01	40.43
**ETC13**	41.20	17.55	15.36	14.70	13.42	**12.94**	13.39	38.28
**ETC22**	81.36	39.93	36.53	34.22	**31.83**	34.22	32.74	78.60
**ETC33**	86.56	43.85	38.58	36.07	**33.43**	34.62	34.94	82.28
**ETC44**	28.48	16.50	15.94	14.75	**13.73**	14.65	14.03	26.82
**ETC55**	26.82	15.37	14.62	13.38	**12.48**	13.30	13.31	24.15
**ETC66**	29.32	16.26	15.60	14.47	**13.16**	13.76	14.44	27.81
**Bulk**	48.21	16.00	11.57	11.88	10.92	**10.62**	11.45	45.85
**Shear**	48.21	13.28	12.35	11.97	**11.19**	11.38	11.57	23.19
**BgMbj**	1.742	0.734	0.529	0.608	**0.491**	0.492	0.519	1.168
**Spillage**	0.533	0.381	0.368	0.374	**0.343**	0.384	0.365	0.491
**SLME**	9.233	6.579	6.914	6.824	**6.425**	6.653	6.646	8.376
**MaxIrM**	485.5	**85.81**	103.7	103.4	96.80	100.2	98.78	406.9
**MinIrM**	65.70	48.82	47.40	44.03	**42.25**	42.60	44.66	62.31
**PMDiEl**	7.227	4.503	4.144	4.777	**3.712**	4.125	3.830	6.243
**PMDi**	8.538	5.001	4.693	4.913	4.421	4.635	**4.277**	7.896
**PMDilo**	2.477	0.892	0.849	0.928	**0.717**	0.804	0.770	2.125
**PMEij**	0.455	**0.306**	0.326	0.345	0.333	0.417	0.388	0.392
**PMDij**	43.66	28.52	28.27	**27.51**	27.54	27.52	28.62	30.07
**Exfoli**	79.49	65.04	66.97	**58.44**	63.77	68.52	62.46	74.47

Supplementary Table 6: MAE of the ElemNet-based source models trained on different materials properties.

Dataset	Property	Size	MAE
OQMD-JARVIS	Formation Energy (eV/atom)	321140	0.0369
	Band Gap (eV)	321140	0.04
	Energy_per_atom (eV/atom)	321140	0.0476
	Stability (eV/atom)	302313	0.0412
	Magnetic Moment (μ_B)	183874	0.1002
	Volume_pa (Å^3)	321140	0.3636

Supplementary Table 7: Prediction performance benchmarking for the prediction task of ”Multi Source Transfer Learning”. The table shows the mean ± standard deviation of MAE for different target properties from 10-fold cross-validation for all models. All the model inputs are based on EF. For each target property, the model with the best mean MAE value is identified by boldfacing its MAE and coloring its cell in grey. Further, the MAEs that were not statistically distinguishable from the best MAE at α=0.05 are also boldfaced (identified using the corrected resampled t-test [1]).

Property	Base	Scratch (SC) Models		Transfer Learning (TL) with different source properties					
		SC: ML(EF)	SC:DL(EF)	Deltae	Bandgap	Volume	Total	Magmom	Stability
**KLU**	19.14±0.17	11.77±0.30	11.63±0.26	**11.28±0.27**	11.74±0.28	**11.39±0.25**	**11.33±0.29**	11.54±0.25	11.39±0.28
**KAA**	5.105±0.080	2.930±0.052	2.983±0.061	**2.811±0.068**	2.960±0.060	2.921±0.068	**2.822±0.066**	2.921±0.064	2.942±0.064
**BgOptb**	0.949±0.012	0.328±0.009	0.249±0.011	**0.229±0.010**	0.261±0.019	0.247±0.010	**0.235±0.010**	**0.229±0.012**	**0.231±0.010**
**Deltae**	0.847±0.013	0.198±0.041	0.134±0.006	**0.121±0.004**	0.152±0.004	0.138±0.005	0.130±0.004	0.141±0.004	0.135±0.005
**Encut**	245.7±2.7	**79.20±2.28**	85.94±2.21	99.29±4.43	88.78±3.82	85.15±2.94	86.21±3.79	86.07±2.94	85.57±3.04
**Ehull**	0.133±0.003	0.069±0.002	0.057±0.002	**0.052±0.002**	0.060±0.003	0.056±0.002	0.055±0.002	0.057±0.003	0.054±0.002
**MagOszi**	1.231±0.021	0.462±0.022	0.423±0.026	**0.385±0.027**	0.418±0.023	0.398±0.028	0.387±0.023	**0.378±0.026**	0.398±0.022
**MagOut**	1.241±0.024	0.457±0.021	0.415±0.023	**0.382±0.023**	0.406±0.015	0.393±0.016	0.387±0.021	**0.374±0.010**	0.409±0.020
**Eps**	3.740±0.052	1.561±0.041	1.464±0.036	**1.305±0.051**	1.462±0.033	1.381±0.047	**1.277±0.026**	1.350±0.032	1.317±0.030
**PPf**	658.0±9.5	543.5±14.6	534.2±13.4	**492.2±17.6**	514.3±14.3	527.1±17.8	**496.4±16.6**	502.6±12.4	**492.2±17.3**
**NPf**	658.2±17.1	556.4±20.6	543.6±19.8	**499.6±22.2**	524.5±19.1	527.6±23.8	**496.8±23.5**	506.9±21.6	**503.9±19.9**
**PEM300K**	1.981±0.045	1.306±0.022	1.272±0.025	1.206±0.035	1.237±0.035	1.233±0.033	**1.170±0.026**	1.207±0.031	1.234±0.023
**NEM300K**	1.981±0.045	1.306±0.022	1.273±0.023	**1.173±0.024**	1.238±0.037	1.243±0.024	**1.169±0.024**	1.201±0.030	1.234±0.023
**PSb**	163.3±2.2	64.84±2.97	61.24±2.33	**54.71±2.13**	56.21±2.65	60.51±2.06	**54.49±2.64**	57.34±2.36	**55.29±2.53**
**NSb**	109.3±1.9	58.22±2.35	54.75±2.23	**49.03±1.99**	50.91±2.67	53.62±2.58	**49.09±2.01**	**50.47±2.61**	**49.82±2.40**
**Meps**	4.928±0.106	2.173±0.067	2.051±0.053	**1.904±0.056**	2.005±0.078	1.984±0.063	**1.864±0.070**	1.916±0.066	**1.897±0.054**
**MaxM**	312.4±17.2	70.24±4.98	70.11±3.99	**64.96±5.07**	74.60±4.98	67.08±5.12	68.03±5.55	67.87±5.55	68.26±5.69
**MinM**	38.52±1.10	24.66±1.50	22.63±1.10	**21.23±1.32**	23.41±1.30	22.29±1.13	**21.20±0.95**	22.78±1.56	**21.27±1.08**
**ETC11**	81.07±2.77	39.96±1.84	36.05±1.76	**32.04±1.32**	35.68±1.49	35.39±1.56	**32.13±1.36**	34.38±1.44	33.95±1.34
**ETC12**	43.39±1.32	19.54±0.78	17.86±0.64	**16.26±0.74**	18.17±0.65	17.00±0.68	**16.58±0.82**	17.14±0.69	**16.59±0.83**
**ETC13**	41.49±1.09	17.09±0.44	14.61±0.69	13.37±0.65	14.91±0.61	13.51±0.59	**12.98±0.57**	13.77±0.62	**13.33±0.40**
**ETC22**	81.08±1.34	40.55±1.08	36.47±1.30	**32.47±1.67**	36.34±1.47	34.17±1.74	**32.34±1.79**	35.52±1.36	**33.46±1.34**
**ETC33**	85.12±2.02	42.77±2.12	38.15±1.64	**33.99±1.65**	38.86±1.99	35.66±1.95	**33.65±1.41**	35.55±1.96	**33.51±1.68**
**ETC44**	29.33±0.87	17.64±0.58	16.65±0.55	**14.10±0.52**	16.57±0.55	14.93±0.55	**14.39±0.52**	15.11±0.68	**14.32±0.43**
**ETC55**	27.54±0.95	15.44±0.71	15.05±0.76	**12.59±0.68**	14.35±0.72	13.72±0.70	13.07±0.75	13.99±0.57	12.86±0.69
**ETC66**	27.52±1.02	15.48±0.64	14.73±0.51	12.77±0.46	14.37±0.53	13.37±0.58	12.88±0.383	13.80±0.50	**12.54±0.46**
**Bulk**	48.39±0.90	16.66±0.40	12.02±0.46	**11.42±0.56**	12.70±0.38	**11.48±0.48**	**11.26±0.41**	**11.40±0.39**	**11.26±0.41**
**Shear**	27.30±7.37	13.72±0.53	12.29±0.46	**10.51±0.46**	11.89±0.53	11.29±0.59	**10.66±0.52**	11.33±0.40	**10.57±0.53**
**BgMbj**	1.843±0.051	0.723±0.041	0.546±0.025	**0.530±0.032**	**0.510±0.012**	0.543±0.026	**0.517±0.019**	**0.519±0.024**	0.543±0.024
**Spillage**	0.496±0.028	0.368±0.019	0.359±0.019	0.362±0.018	**0.351±0.017**	0.357±0.013	0.367±0.019	0.368±0.021	**0.335±0.020**
**SLME**	9.419±0.381	6.959±0.404	6.775±0.446	6.581±0.353	**6.526±0.358**	6.779±0.381	6.696±0.422	**6.430±0.347**	**6.434±0.305**
**MaxIrM**	445.5±35.7	**96.98±12.30**	99.91±7.16	94.18±8.46	106.51±7.66	99.34±2.96	**88.88±4.93**	94.69±9.05	101.30±6.04
**MinIrM**	66.00±3.70	46.74±4.93	42.16±3.52	42.08±1.77	41.99±2.97	41.05±2.97	**39.73±3.59**	44.60±2.77	**37.88±2.93**
**PMDiEl**	6.814±0.471	4.363±0.555	4.116±0.408	4.128±0.511	4.532±0.567	**3.880±0.470**	**3.903±0.383**	4.372±0.505	**3.788±0.423**
**PMDi**	8.124±0.410	4.937±0.517	4.714±0.390	**4.498±0.532**	5.246±0.614	4.566±0.347	**4.225±0.391**	4.963±0.460	**4.327±0.460**
**PMDilo**	2.630±0.218	0.888±0.154	0.820±0.111	**0.774±0.111**	**0.815±0.116**	**0.819±0.157**	**0.778±0.109**	**0.813±0.096**	**0.796±0.104**
**PMEij**	0.479±0.087	0.353±0.066	**0.324±0.061**	0.375±0.067	**0.314±0.047**	**0.314±0.055**	0.361±0.087	**0.320±0.065**	**0.322±0.055**
**PMDij**	56.90±20.73	38.55±23.36	**37.36±22.69**	**38.01±23.54**	38.36±23.41	84.32±50.63	**37.99±23.49**	**38.08±23.56**	**38.06±23.15**
**Exfoli**	57.46±11.29	44.63±13.58	**41.46±13.69**	**44.03±6.79**	**44.29±11.30**	55.45±13.85	59.91±13.15	48.04±9.39	**49.20±6.42**

Supplementary Table 8: The table shows the best performing SC and TL models from Supplementary Table 7 that are used on the test set to get the results shown in Table 3. The notations for specific models used in this work are described in Table 1. For TL models, the source property used to get the best model and layer from which features were extracted (in case of FeatExtr method) is also mentioned. The model that gave a better test MAE (as in Table 3) is boldfaced.

**Property**	**Best Performing SC Model**	**Best Performing TL Model**
KLU	SC:DL(EF)	**TL:ML(FeatExtr [Layer2]) { Deltae }**
KAA	SC:ML(EF)	**TL:DL(FeatExtr [Layer3]) { Deltae }**
BgOptb	SC:DL(EF)	**TL:ModFineTune { Deltae }**
Deltae	SC:DL(EF)	**TL:FineTune { Deltae }**
Encut	**SC:ML(EF)**	TL:DL(FeatExtr [Layer1]) { Volume }
Ehull	SC:DL(EF)	**TL:FineTune { Deltae }**
Magoszi	SC:DL(EF)	**TL:FineTune { Magmom }**
Magout	SC:DL(EF)	**TL:ModFineTune { Magmom }**
Eps	SC:DL(EF)	**TL:DL(FeatExtr [Layer2]) { Total }**
PPF	SC:DL(EF)	**TL:DL(FeatExtr [Layer3]) { Stab }**
NPF	SC:DL(EF)	**TL:DL(FeatExtr [Layer2]) { Total }**
Pem300k	SC:DL(EF)	**TL:DL(FeatExtr [Layer2]) { Total }**
Nem300k	SC:DL(EF)	**TL:DL(FeatExtr [Layer2]) { Total }**
PSB	SC:DL(EF)	**TL:DL(FeatExtr [Layer2]) { Total }**
NSB	SC:DL(EF)	**TL:DL(FeatExtr [Layer2]) { Deltae }**
Meps	SC:DL(EF)	**TL:DL(FeatExtr [Layer2]) { Total }**
MaxM	SC:DL(EF)	**TL:ModFineTune { Deltae }**
MinM	SC:DL(EF)	**TL:DL(FeatExtr [Layer2]) { Total }**
ETC11	SC:DL(EF)	**TL:DL(FeatExtr [Layer3]) { Deltae }**
ETC12	SC:DL(EF)	**TL:DL(FeatExtr [Layer3]) { Deltae }**
ETC13	SC:DL(EF)	**TL:DL(FeatExtr [Layer2]) { Total }**
ETC22	SC:DL(EF)	**TL:DL(FeatExtr [Layer2]) { Total }**
ETC33	SC:DL(EF)	**TL:DL(FeatExtr [Layer3]) { Stab }**
ETC44	SC:DL(EF)	**TL:DL(FeatExtr [Layer3]) { Deltae }**
ETC55	SC:DL(EF)	**TL:DL(FeatExtr [Layer3]) { Deltae }**
ETC66	SC:DL(EF)	**TL:DL(FeatExtr [Layer3]) { Stab }**
BulkKV	SC:DL(EF)	**TL:ModFineTune { Total }**
ShearGV	SC:DL(EF)	**TL:DL(FeatExtr [Layer3]) { Deltae }**
BgMbj	SC:DL(EF)	**TL:DL(FeatExtr [Layer2]) { Bandgap }**
Spillage	SC:DL(EF)	**TL:DL(FeatExtr [Layer2]) { Stab }**
SLME	SC:DL(EF)	**TL:DL(FeatExtr [Layer3]) { Magmom }**
MaxIrM	SC:ML(EF)	**TL:ModFineTune { Total }**
MinIrM	SC:DL(EF)	**TL:DL(FeatExtr [Layer2]) { Stab }**
PMDiEl	SC:DL(EF)	**TL:DL(FeatExtr [Layer1]) { Stab }**
PMDi	SC:DL(EF)	**TL:DL(FeatExtr [Layer2]) { Total }**
PMDiIo	SC:DL(EF)	**TL:DL(FeatExtr [Layer2]) { Deltae }**
PMEij	SC:DL(EF)	**TL:DL(FeatExtr [Layer3]) { Bandgap }**
PMDij	SC:DL(EF)	**TL:ML(FeatExtr [Layer1]) { Total }**
Exfoli	SC:DL(EF)	**TL:ML(FeatExtr [Layer3]) { Deltae }**

Supplementary Table 9: Prediction performance benchmarking for the prediction task of ”Multi Source Transfer Learning”. The table shows the mean $\pm$ standard deviation of MAE for different target properties from 10-fold cross-validation for all models. The SC models are allowed to use PA as input as well, while TL models only use EF-based inputs. For each target property, the model with the best mean MAE value is identified by boldfacing its MAE and coloring its cell in grey. Further, the MAEs that were not statistically distinguishable from the best MAE at α=0.05 are also boldfaced (identified using the corrected resampled t-test [1]).

Property	Base	Scratch (SC) Models				Transfer Learning (TL) with different source properties					
		SC: ML(EF)	SC: ML(PA)	SC:DL(EF)	SC: DL(PA)	Deltae	Bandgap	Volume	Total	Magmom	Stability
KLU	19.14±0.17	11.77±0.30	**10.79±0.24**	11.63±0.26	11.40±0.27	11.28±0.27	11.74±0.28	11.39±0.25	11.33±0.29	11.54±0.25	11.39±0.28
KAA	5.105±0.080	2.930±0.052	**2.667±0.039**	2.983±0.061	2.837±0.045	2.811±0.068	2.960±0.060	2.921±0.068	2.822±0.066	2.921±0.064	2.942±0.064
BgOptb	0.949±0.012	0.328±0.009	0.250±0.008	0.249±0.011	0.250±0.008	**0.229±0.010**	0.261±0.019	0.247±0.010	**0.235±0.010**	**0.229±0.012**	**0.231±0.010**
Deltae	0.847±0.013	0.198±0.041	0.157±0.004	0.134±0.006	0.166±0.002	**0.121±0.004**	0.152±0.004	0.138±0.005	0.130±0.004	0.141±0.004	0.135±0.005
Encut	245.7±2.7	**79.20±2.28**	83.26±2.48	85.94±2.21	89.38±3.26	99.29±4.43	88.78±3.82	85.15±2.94	86.21±3.79	86.07±2.94	85.57±3.04
Ehull	0.133±0.003	0.069±0.002	0.066±0.002	0.057±0.002	0.059±0.002	**0.052±0.002**	0.060±0.003	0.056±0.002	0.055±0.002	0.057±0.003	0.054±0.002
MagOszi	1.231±0.021	0.462±0.022	0.464±0.015	0.423±0.026	0.502±0.098	**0.385±0.027**	0.418±0.023	0.398±0.028	0.387±0.023	**0.378±0.026**	0.398±0.022
MagOut	1.241±0.024	0.457±0.021	0.465±0.024	0.415±0.023	0.546±0.149	**0.382±0.023**	0.406±0.015	0.393±0.016	0.387±0.021	**0.374±0.010**	0.409±0.020
Eps	3.740±0.052	1.561±0.041	1.298±0.026	1.464±0.036	1.472±0.037	**1.305±0.051**	1.462±0.033	1.381±0.047	**1.277±0.026**	1.350±0.032	1.317±0.030
PPf	658.0±9.5	543.5±14.6	**493.5±14.1**	534.2±13.4	507.0±16.7	**492.2±17.6**	514.3±14.3	527.1±17.8	**496.4±16.6**	502.6±12.4	**492.2±17.3**
NPf	658.2±17.1	556.4±20.6	**503.3±15.7**	543.6±19.8	517.6±17.5	**499.6±22.2**	524.5±19.1	527.6±23.8	**496.8±23.5**	506.9±21.6	**503.9±19.9**
PEM300K	1.981±0.045	1.306±0.022	**1.119±0.028**	1.272±0.025	1.231±0.016	1.206±0.035	1.237±0.035	1.233±0.033	1.170±0.026	1.207±0.031	1.234±0.023
NEM300K	1.981±0.045	1.306±0.022	**1.119±0.028**	1.273±0.023	1.230±0.017	1.173±0.024	1.238±0.037	1.243±0.024	1.169±0.024	1.201±0.030	1.234±0.023
PSb	163.3±2.2	64.84±2.97	**55.11±1.97**	61.24±2.33	56.68±3.04	**54.71±2.13**	56.21±2.65	60.51±2.06	**54.49±2.64**	57.34±2.36	**55.29±2.53**
NSb	109.3±1.9	58.22±2.35	**48.33±1.09**	54.75±2.23	52.18±2.16	**49.03±1.99**	50.91±2.67	53.62±2.58	**49.09±2.01**	50.47±2.61	**49.82±2.40**
Meps	4.928±0.106	2.173±0.067	1.938±0.075	2.051±0.053	2.085±0.063	**1.904±0.056**	2.005±0.078	1.984±0.063	**1.864±0.070**	1.916±0.066	**1.897±0.054**
MaxM	312.4±17.2	70.24±4.98	**61.60±4.73**	70.11±3.99	65.48±5.39	64.96±5.07	74.60±4.98	67.08±5.12	68.03±5.55	67.87±5.55	68.26±5.69
MinM	38.52±1.10	24.66±1.50	25.44±1.35	22.63±1.10	**21.71±1.10**	**21.23±1.32**	23.41±1.30	22.29±1.13	**21.20±0.95**	22.78±1.56	**21.27±1.08**
ETC11	81.07±2.77	39.96±1.84	33.09±1.05	36.05±1.76	35.49±1.40	**32.04±1.32**	35.68±1.49	35.39±1.56	**32.13±1.36**	34.38±1.44	33.95±1.34
ETC12	43.39±1.32	19.54±0.78	**16.60±0.63**	17.86±0.64	17.60±0.60	**16.26±0.74**	18.17±0.65	17.00±0.68	**16.58±0.82**	17.14±0.69	**16.59±0.83**
ETC13	41.49±1.09	17.09±0.44	**13.20±0.45**	14.61±0.69	14.10±0.45	13.37±0.65	14.91±0.61	13.51±0.59	**12.98±0.57**	13.77±0.62	**13.33±0.40**
ETC22	81.08±1.34	40.55±1.08	33.37±1.10	36.47±1.30	35.90±1.47	**32.47±1.67**	36.34±1.47	34.17±1.74	**32.34±1.79**	35.52±1.36	**33.46±1.34**
ETC33	85.12±2.02	42.77±2.12	**34.58±1.60**	38.15±1.64	36.92±1.69	**33.99±1.65**	38.86±1.99	35.66±1.95	**33.65±1.41**	35.55±1.96	**33.51±1.68**
ETC44	29.33±0.87	17.64±0.58	14.85±0.37	16.65±0.55	15.99±0.45	**14.10±0.52**	16.57±0.55	14.93±0.55	**14.39±0.52**	15.11±0.68	**14.32±0.43**
ETC55	27.54±0.95	15.44±0.71	13.19±0.39	15.05±0.76	14.30±0.53	**12.59±0.68**	14.35±0.72	13.72±0.70	13.07±0.75	13.99±0.57	12.86±0.69
ETC66	27.52±1.02	15.48±0.64	12.97±0.57	14.73±0.51	14.13±0.47	12.77±0.46	14.37±0.53	13.37±0.58	12.88±0.38	13.80±0.50	**12.54±0.46**
Bulk	48.39±0.90	16.66±0.40	12.35±0.43	12.02±0.46	12.68±0.51	**11.42±0.56**	12.70±0.38	**11.48±0.48**	**11.26±0.41**	**11.40±0.39**	**11.26±0.41**
Shear	27.30±7.37	13.72±0.53	11.24±0.42	12.29±0.46	12.12±0.50	**10.51±0.46**	11.89±0.53	11.29±0.59	**10.66±0.52**	11.33±0.40	**10.57±0.53**
BgMbj	1.843±0.051	0.723±0.041	0.572±0.026	0.546±0.025	0.577±0.015	**0.530±0.032**	**0.510±0.012**	0.543±0.026	**0.517±0.019**	**0.519±0.024**	0.543±0.024
Spillage	0.496±0.028	0.368±0.019	0.368±0.014	0.359±0.019	**0.348±0.020**	0.362±0.018	**0.351±0.017**	0.357±0.013	0.367±0.019	0.368±0.021	**0.335±0.020**
SLME	9.419±0.381	6.959±0.404	6.810±0.190	6.775±0.446	6.862±0.444	6.581±0.353	**6.526±0.358**	6.779±0.381	6.696±0.422	**6.430±0.347**	**6.434±0.305**
MaxIrM	445.5±35.7	**96.98±12.30**	**89.17±9.24**	99.91±7.16	**87.36±8.29**	**94.18±8.46**	106.51±7.66	99.34±2.96	**88.88±4.93**	**94.69±9.05**	101.30±6.04
MinIrM	66.00±3.70	46.74±4.93	**38.34±2.44**	42.16±3.52	**39.17±3.23**	42.08±1.77	41.99±2.97	41.05±2.97	**39.73±3.59**	44.60±2.77	**37.88±2.93**
PMDiEl	6.814±0.471	4.363±0.555	4.398±0.567	4.116±0.408	**3.954±0.560**	4.128±0.511	4.532±0.567	**3.880±0.470**	**3.903±0.383**	4.372±0.505	**3.788±0.423**
PMDi	8.124±0.410	4.937±0.517	4.789±0.468	4.714±0.390	4.540±0.633	**4.498±0.532**	5.246±0.614	4.566±0.347	**4.225±0.391**	4.963±0.460	**4.327±0.460**
PMDilo	2.630±0.218	0.888±0.154	0.877±0.134	0.820±0.111	0.870±0.161	**0.774±0.111**	**0.815±0.116**	**0.819±0.157**	**0.778±0.109**	**0.813±0.096**	**0.796±0.104**
PMEij	0.479±0.087	0.353±0.066	0.442±0.070	**0.324±0.061**	0.345±0.067	0.375±0.067	**0.314±0.047**	**0.314±0.055**	0.361±0.087	**0.320±0.065**	**0.322±0.055**
PMDij	56.90±20.73	38.55±23.36	38.91±22.94	**37.36±22.69**	**34.27±22.57**	38.01±23.54	38.36±23.41	84.32±50.63	37.99±23.49	38.08±23.56	38.06±23.15
Exfoli	57.46±11.29	**44.63±13.58**	**43.42±9.78**	**41.46±13.69**	**39.82±14.46**	**44.03±6.79**	**44.29±11.30**	55.45±13.85	59.91±13.15	48.04±9.39	49.20±6.42

Supplementary Table 10: The table shows the best performing SC and TL models from Supplementary Table 9 that are used on the test set to get the results shown in Table 4. The notations for specific models used in this work are described in Table 1. For TL models, the source property used to get the best model and layer from which features was extracted (in case of FeatExtr method) is also mentioned. The model that gave a better test MAE (as in Table 4) is boldfaced.

**Property**	**Best Performing SC Model**	**Best Performing TL Model**
KLU	**SC:ML(PA)**	TL:ML(FeatExtr [Layer2]) { Deltae }
KAA	**SC:ML(PA)**	TL:DL(FeatExtr [Layer3]) { Deltae }
BgOptb	SC:DL(EF)	**TL:ModFineTune { Deltae }**
Deltae	SC:DL(EF)	**TL:FineTune { Deltae }**
Encut	**SC:ML(EF)**	TL:DL(FeatExtr [Layer1]) { Volume }
Ehull	SC:DL(EF)	**TL:FineTune { Deltae }**
Magoszi	SC:DL(EF)	**TL:FineTune { Magmom }**
Magout	SC:DL(EF)	**TL:ModFineTune { Magmom }**
Eps	SC:ML(PA)	**TL:DL(FeatExtr [Layer2]) { Total }**
PPF	SC:ML(PA)	**TL:DL(FeatExtr [Layer3]) { Stab }**
NPF	**SC:ML(PA)**	TL:DL(FeatExtr [Layer2]) { Total }
Pem300k	**SC:ML(PA)**	TL:DL(FeatExtr [Layer2]) { Total }
Nem300k	**SC:ML(PA)**	TL:DL(FeatExtr [Layer2]) { Total }
PSB	**SC:ML(PA)**	TL:DL(FeatExtr [Layer2]) { Total }
NSB	**SC:ML(PA)**	TL:DL(FeatExtr [Layer2]) { Deltae }
Meps	SC:ML(PA)	**TL:DL(FeatExtr [Layer2]) { Total }**
MaxM	**SC:ML(PA)**	TL:ModFineTune { Deltae }
MinM	SC:DL(PA)	**TL:DL(FeatExtr [Layer2]) { Total }**
ETC11	SC:ML(PA)	**TL:DL(FeatExtr [Layer3]) { Deltae }**
ETC12	**SC:ML(PA)**	TL:DL(FeatExtr [Layer3]) { Deltae }
ETC13	SC:ML(PA)	**TL:DL(FeatExtr [Layer2]) { Total }**
ETC22	SC:ML(PA)	**TL:DL(FeatExtr [Layer2]) { Total }**
ETC33	SC:ML(PA)	**TL:DL(FeatExtr [Layer3]) { Stab }**
ETC44	SC:ML(PA)	**TL:DL(FeatExtr [Layer3]) { Deltae }**
ETC55	SC:ML(PA)	**TL:DL(FeatExtr [Layer3]) { Deltae }**
ETC66	SC:ML(PA)	**TL:DL(FeatExtr [Layer3]) { Stab }**
BulkKV	SC:DL(EF)	**TL:ModFineTune { Total }**
ShearGV	SC:ML(PA)	**TL:DL(FeatExtr [Layer3]) { Deltae }**
BgMbj	SC:DL(EF)	**TL:DL(FeatExtr [Layer2]) { Bandgap }**
Spillage	SC:DL(PA)	**TL:DL(FeatExtr [Layer2]) { Stab }**
SLME	SC:DL(EF)	**TL:DL(FeatExtr [Layer3]) { Magmom }**
MaxIrM	**SC:DL(PA)**	TL:ModFineTune { Total }
MinIrM	**SC:ML(PA)**	TL:DL(FeatExtr [Layer2]) { Stab }
PMDiEl	SC:DL(PA)	**TL:DL(FeatExtr [Layer1]) { Stab }**
PMDi	SC:DL(PA)	**TL:DL(FeatExtr [Layer2]) { Total }**
PMDiIo	SC:DL(EF)	**TL:DL(FeatExtr [Layer2]) { Deltae }**
PMEij	SC:DL(EF)	**TL:DL(FeatExtr [Layer3]) { Bandgap }**
PMDij	SC:DL(PA)	**TL:ML(FeatExtr [Layer1]) { Total }**
Exfoli	SC:DL(PA)	**TL:ML(FeatExtr [Layer3]) { Deltae }**

Supplementary Table 11: Prediction performance benchmarking for the prediction task of ”Transfer Learning on Experimental Data”. The table shows the mean ± standard deviation of MAE for different target properties from 100-fold cross-validation for all models. The SC models are allowed to use PA as input as well, while TL models only use EF-based inputs. For each target property, the model with the best mean MAE value is identified by boldfacing its MAE and coloring its cell in grey. Further, the MAEs that were not statistically distinguishable from the best MAE at α=0.05 are also boldfaced (identified using the corrected resampled t-test [1]).

**Property (Units):Size**	**Scratch (SC) Models**						**Transfer Learning (TL) Models**			
	**SC:ML(EF)**	**SC:ML(PA)**	**AutoML(EF)**	**AutoML(PA)**	**SC:DL(EF)**	**SC:DL(PA)**	**TL:ML(FeatExtr)**	**TL:DL(FeatExtr)**	**TL:FineTune**	**TL:ModFineTune**
**Formation Enthalpy (eV/atom):1643**	0.1902 ±0.0593	0.1586 ±0.0482	0.2465 ±0.0803	0.1186 ±0.0415	0.1095 ±0.0399	0.1115 ±0.0413	0.0987 ±0.0305	**0.0785 ±0.0290**	**0.0745 ±0.0306**	0.0879 ±0.0322
**Band Gap (eV):4920**	0.5483 ±0.1572	0.4560 ±0.1282	0.4938 ±0.1370	0.3466 ±0.0910	0.3323 ±0.1237	0.3179 ±0.1009	0.4350 ±0.1240	**0.2638 ± 0.1000**	0.3243 ±0.1007	0.3163 ±0.0985

Supplementary Table 12: The table shows the 95th percentile of the distribution of the absolute error (Q_{95}) of the best models used in Tables 2-5 on the test set for each of the target materials properties.

**Property**	Q_{95} for Best Model in Tables 2 and 5		Q_{95} for Best Model in Tables 3 and 5		Q_{95} for Best Model in Tables 4 and 5	
	**Best SC Model**	**Best TL Model**	**Best SC Model**	**Best TL Model**	**Best SC Model**	**Best TL Model**
KLU	35.27	**32.90**	35.27	**34.12**	**29.24**	34.12
KAA	9.680	**8.166**	9.680	**8.882**	**8.210**	8.882
BgOptb	1.576	**1.283**	1.576	**1.283**	1.576	**1.283**
Deltae	0.514	**0.445**	0.514	**0.445**	0.514	**0.445**
Encut	**315.0**	351.2	**315.0**	357.4	**315.0**	357.4
Ehull	0.342	**0.298**	0.342	**0.298**	0.342	**0.298**
Magoszi	2.928	**2.674**	2.928	**2.674**	2.928	**2.674**
Magout	2.618	**2.273**	2.618	**2.273**	2.618	**2.273**
Eps	4.976	**4.284**	4.976	**4.626**	**4.051**	4.626
PPF	1801.9	**1720.3**	1801.9	**1741.8**	**1415.1**	1741.8
NPF	1951.6	**1718.9**	1951.6	**1765.9**	**1413.3**	1765.9
Pem300k	**4.145**	4.195	4.145	**4.113**	**3.449**	4.113
Nem300k	4.197	**4.172**	4.197	**4.129**	**3.449**	4.129
PSB	325.8	**226.5**	325.8	**314.1**	**218.7**	314.1
NSB	371.7	**371.6**	371.7	**371.6**	**167.6**	371.6
Meps	7.312	**6.521**	7.312	**6.796**	**6.003**	6.796
MaxM	249.4	**214.0**	249.4	**214.0**	**213.8**	214.0
MinM	174.5	**167.4**	174.5	**131.2**	174.5	**131.2**
ETC11	126.7	**114.8**	126.7	**118.7**	119.0	**118.7**
ETC12	63.72	**63.55**	63.72	**61.73**	**51.37**	61.73
ETC13	50.93	**46.46**	50.93	**46.96**	47.06	**46.96**
ETC22	124.8	**120.7**	124.8	**118.8**	121.0	**118.8**
ETC33	122.3	**118.1**	122.3	**115.3**	120.0	**115.3**
ETC44	57.99	**54.29**	57.99	**50.75**	50.98	**50.75**
ETC55	50.31	**38.79**	50.31	**38.78**	40.57	**38.78**
ETC66	51.26	**51.18**	51.26	**45.63**	47.17	**45.63**
BulkKV	40.96	**40.79**	40.96	**37.34**	40.96	**37.34**
ShearGV	43.96	**39.20**	43.96	**39.54**	43.96	**39.54**
BgMbj	2.075	**2.010**	2.075	**2.066**	2.075	**2.066**
Spillage	1.508	**1.353**	1.508	**1.429**	1.568	**1.429**
SLME	21.81	**19.60**	21.81	**19.28**	21.81	**19.28**
MaxIrM	487.5	**425.3**	487.5	**442.1**	**271.1**	442.1
MinIrM	184.2	**153.2**	184.2	**172.2**	**128.1**	172.2
PMDiEl	**11.90**	13.95	11.90	12.80	13.41	**12.80**
PMDi	11.65	**11.34**	11.65	**11.34**	14.77	**11.34**
PMDiIo	**2.786**	3.245	2.786	**2.662**	2.786	**2.662**
PMEij	1.734	**1.633**	1.734	**1.287**	1.734	**1.287**
PMDij	102.16	**93.21**	102.16	**98.11**	129.7	**98.11**
Exfoli	240.5	**196.9**	240.5	**184.7**	229.0	**184.7**
Expt Deltae	0.264	**0.196**	0.264	**0.196**	0.264	**0.196**
Expt Band Gap	2.238	**1.667**	2.238	**1.667**	2.304	**1.667**

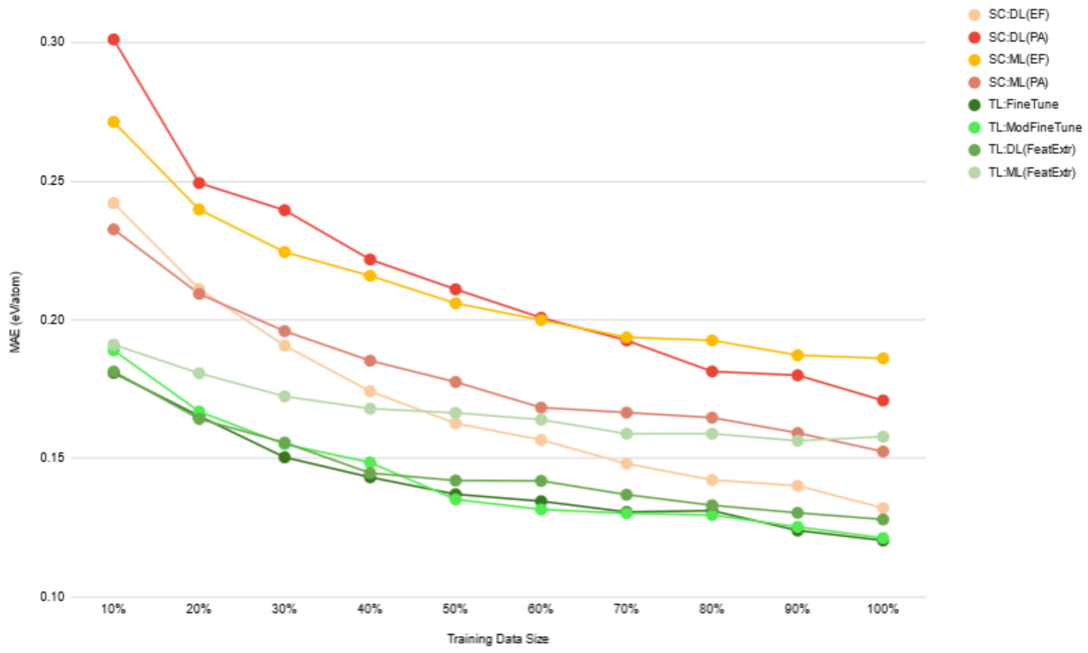

Supplementary Figure 1: Training curve for prediction accuracy of target formation energy for different training data sizes on a fixed test set.

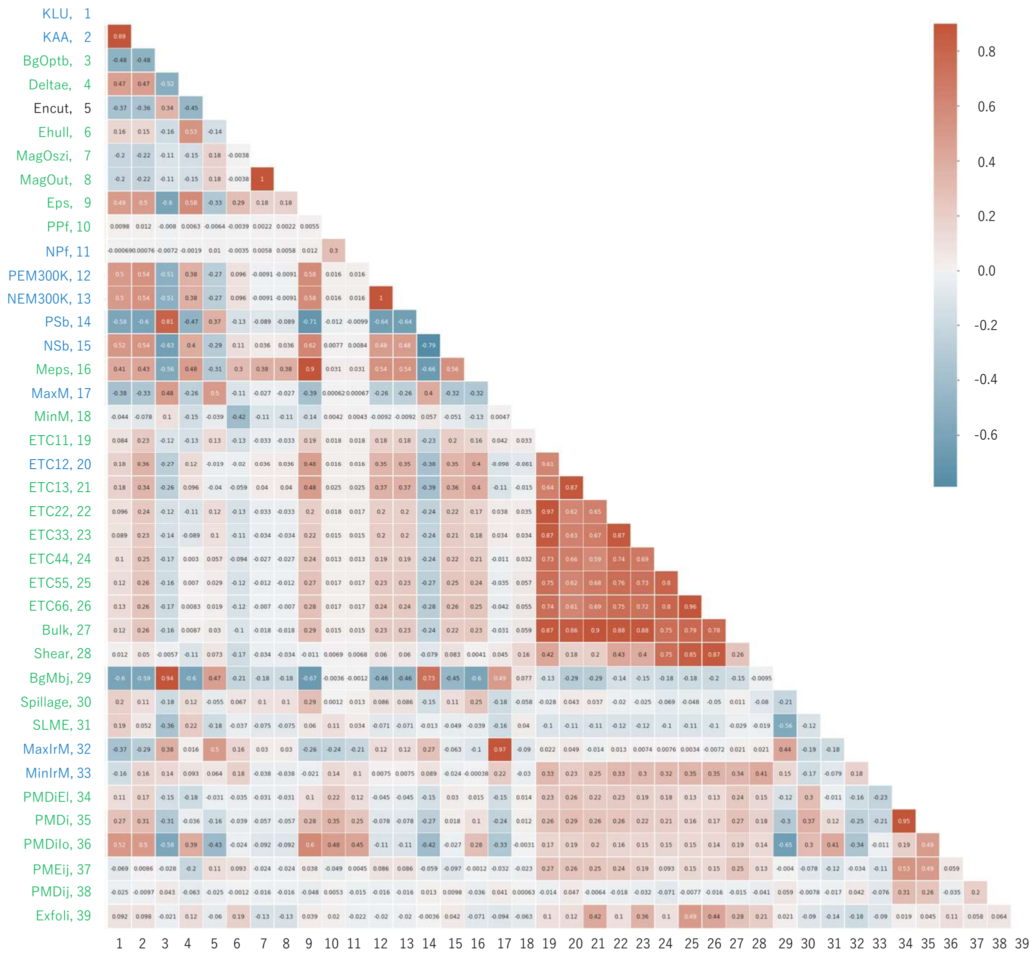

Supplementary Figure 2: Correlation between the Target Properties. Positive (negative) correlation is depicted by red (blue) color and can be seen pair-wise between properties. The materials properties indicated with green (blue) color represents those for which TL models performs better than SC models when both EF and PA (only EF) are used as an input for SC models. Those indicated with black color represents those properties for which TL did not perform better in any case.

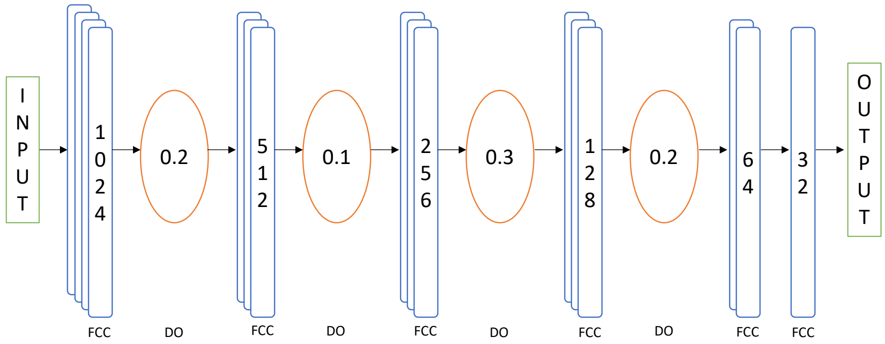

Supplementary Figure 3: Architecture of the DL model (ElemNet) used in this work. The stacked structure represents same layer connected continuously. FCC and DO represents the fully connected layer and Dropout respectively. Dropout is not applied during the validation/testing phase for improved performance.

Supplementary References

1. Nadeau, C. & Bengio, Y. Inference for the generalization error. *Machine learning* **52**, 239–281 (2003).
